# Variable data structures and customized deep learning surrogates for computationally efficient and reliable characterization of buried objects

**DOI:** 10.1038/s41598-024-65996-0

**Published:** 2024-06-28

**Authors:** Reyhan Yurt, Hamid Torpi, Ahmet Kizilay, Slawomir Koziel, Peyman Mahouti

**Affiliations:** 1 Kırşehir Department of Electrical and Electronics Engineering, Kırşehir Ahi Evran University, 40100 Kırşehir, Turkey; 2 Department of Electronics and Communication Engineering, Yıldız Technical University, 34220 İstanbul, Turkey; 3 Department of Engineering, Engineering Optimization and Modeling Center, Reykjavik University, Menntavegur 1, 101 Reykjavik, Iceland; 4 Faculty of Electronics, Telecommunications and Informatics, Gdansk University of Technology, Narutowicza 11/12, 80-233 Gdansk, Poland

**Keywords:** Artificial intelligence, Buried object characterization, Deep regression network, Ground penetrating radar (GPR), Surrogate modeling, Time frequency spectrogram, Electrical and electronic engineering, Computational science

## Abstract

In this study, in order to characterize the buried object via deep-learning-based surrogate modeling approach, 3-D full-wave electromagnetic simulations of a GPR model have been used. The task is to independently predict characteristic parameters of a buried object of diverse radii allocated at different positions (depth and lateral position) in various dispersive subsurface media. This study has analyzed variable data structures (raw B-scans, extracted features, consecutive A-scans) with respect to computational cost and accuracy of surrogates. The usage of raw B-scan data and the applications for processing steps on B-scan profiles in the context of object characterization incur high computational cost so it can be a challenging issue. The proposed surrogate model referred to as the deep regression network (DRN) is utilized for time frequency spectrogram (TFS) of consecutive A-scans. DRN is developed with the main aim being computationally efficient (about 13 times acceleration) compared to conventional network models using B-scan images (2D data). DRN with TFS is favorably benchmarked to the state-of-the-art regression techniques. The experimental results obtained for the proposed model and second-best model, CNN-1D show mean absolute and relative error rates of 3.6 mm, 11.8 mm and 4.7%, 11.6% respectively. For the sake of supplementary verification under realistic scenarios, it is also applied for scenarios involving noisy data. Furthermore, the proposed surrogate modeling approach is validated using measurement data, which is indicative of suitability of the approach to handle physical measurements as data sources.

## Introduction

The analysis of subsurface medium for identification of buried objects is an important endeavor with numerous practical applications and consequences. One of these is identification of landmines or explosive devices, which pose serious threats to human life, both in the military and civilian context^1–3^. In addition, identification of pipes, rebars, cables (electric, phone, optical fiber cables) has a great significance for (non-destructive) structural evaluation, subsurface mapping, as well as building inspection^4–9^. At the same time, in many scenarios, the mere detection of the object is insufficient, and more detailed information needs to be acquired such as object location, orientation, and size. Thus, the estimation of object's characteristic parameters in terms of the radius, the depth and the lateral position are significant aspects of buried object characterization.

One of the most popular approaches to buried object identification is the employment of ground penetrating radars (GPRs). GPR systems allow for accounting for anomalies in the underground medium, as well as analyzing the subsurface reflections mechanisms due to the presence of a buried object. GPR has been widely applied in remote sensing, and it is considered a fast, convenient, efficient and reliable tool of choice for underground investigations and identification of buried objects based on the analysis of the reflected signals^10–12^.

The working principle of GPR-based systems is transmission and reception of electromagnetic waves using antennas. The key elements in a GPR system are transmitter and receiver antennas in bistatic configuration for sending and receiving time-domain or frequency signals. Herein, one antenna in monostatic configuration^13,14^, which is a conventional C-Band (4–8 GHz) horn antenna^14^ is moved along a synthetic aperture to scan the underground and to enable buried object characterization with regards to localization and radius estimation by using data-driven surrogate models. The scanning process is realized by moving the antenna along a scanning path on the upper surface of the ground (referred to as subsurface medium). The signal received at one point is referred to as an A-scan, which is 1D time-varying amplitude signal. After the scanning process along the entire path has been completed, all received A-scan signals are merged into a B-scan (2D data) image^15^. For the purpose of GPR-based identification, the buried object in subsurface medium is often defined as a perfect electrical conductor (PEC) and of a cylindrical shape like a rebar, a pipeline or a wire (energy, signal or optical cable). It produces a hyperbolic signature (pattern) in B-scan^6^. Identification of hyperbolic pattern’s is the widely applied technique for object detection, determining its position, and prediction of the object dimension (radius or diameter for a cylindrical shape) by using numerical, analytical and artificial intelligence (AI) methods^3,7,16–24^. In the literature, numerous modeling approaches with various data types such as B-scan images, extracted features and hyperbolic signatures have been developed and applied to characterize reflected signals of the object buried in the subsurface medium. B-scans have led to successful results but also incur considerable computational burden related to a construction of respective surrogate models, which is a limitation of the approaches that rely on B-scan images. To mitigate this and other issues of GPR-based object identification, several approaches have been developed that employ a variety of signal pre-processing methods as well as efficient surrogate modeling techniques to represent the data acquired by the system.

In order to examine the reflected signals owing to the presence of an object, and to extract object-related features such as shape, depth, lateral position, and size, specialized techniques are applied to the received signals before launching the object identification process. These are known as pre-processing methods. One of the most common pre-processing approaches is background subtraction, which removes direct wave and the air-ground surface echoes. This affects to a great extent the performance of characterization procedures in terms of predicting geometrical and physical parameters, material type and object shape^15–17,19–22,24–26^. Other clutter reduction (pre-processing) methods include principal component analysis (PCA)^15,27–29^, morphological component analysis (MCA)^30^, singular value decomposition (SVD)^15,29^, independent component analysis (ICA)^15,29^, as well as ICA with multifractal spectrum^31^. In addition to clutter removing, another pre-processing application of PCA is feature extraction, which aims at dimensionality reduction of the B-scan image (2-D data)^32,33^. The latter is beneficial from the point of view of representing data using surrogate modeling methods. In particular, it improves computational efficiency of object characterization process by diminishing the expenses associated with a construction of the underlying surrogate model. Furthermore, feature extraction techniques are applied for material classification of the object, using geometrical features such as minor and major axes, along with principal components (PCs); also statistical features (SFs) such as mean, variance and kurtosis, etc. In conjunction to these, support vector machines (SVM) and neural network (NN) classifiers^32^ are often employed as well. Similarly, identification of buried materials has been carried out using SVM and extracted statistical features (SFs) from fractional domain-envelope curve^34^. In another study, statistical features (variance, deviation, kurtosis and skewness) extracted from the reflected signals in noisy environment for classification of the material type have been compared with the spectral features^8^. Also, after pre-processing B-scan data hyperbola is fitted by using segmentation curvature features^35^ and other features have been extracted for object classification. Furthermore, the features extracted from a reflected wave in time and frequency domain have been used for discrimination of air, water, conductor materials in a classification approach and estimation of depth and radius in a regression approach by using k-Nearest Neighbor (k-NN) model^36^.

Dou et al.^17^, employed a Column-Connection-Clustering (C3) algorithm after pre-processing of B-scans to identify the hyperbola; the C3 outputs are classified for hyperbolic identification using the NN model^17^. In addition to investigating hyperbolic signatures after the pre-processing operations, hyperbolic extraction through deep learning based model, Single Shot Multibox Detector (SSD)^6^ has been utilized for identification of buried objects and determining their positions. In another study^16^, hyperbolic curve identification has been carried out in the pre-processed B-scan image for object detection, classification of material type and localization with the help of genetic algorithms (GAs). In^18^, a classification-based machine learning framework incorporating wavelet scattering and SVM in a cascaded structure has been proposed for pipeline identification, localization and diameter estimation from reconstructed GPR profiles. A substituted hyperbolic summation method using hyperbola has been developed to obtain focused GPR B-scan image^14^. In another study, after removing subsurface reflections the extracted hyperbola from the B-scan profiles in terms of a vector including amplitude and time generated by the Hilbert transform (HT) is utilized to arrange inputs of a NN in cascaded architecture for buried object characterization^9^. Furthermore, the hyperbolic signatures are taken out of the background subtracted B-scan images by using linear regression algorithm, and used for inputs of deep learning based Modified-Multilayer-Perceptron (M2LP)^37^ surrogate model to obtain characteristic parameters of the object.

For buried object recognition by means of B-scan data, deep learning models (DL) have demonstrated considerable achievement, particularly convolutional neural network (CNN) frameworks^3,7,19,21,22,25,38^. In a framework including CNN and LSTM (long-short term memory) in a cascaded configuration^3^, 3D GPR data created with cross and along axes are investigated for recognition of buried explosive object and discrimination between target and non-target alarms. In another investigation aiming at detection of a cylindrical object^7^, CNN is used together with the LSTM network. Also, objects are categorized into nine varied diameters in the extracted hyperbola sections using B-scan data^7^ created through gprMax simulation tool^39,40^. In a study^25^, dedicated deep learning framework involving CNN model has been employed along with the SVM classifier rather than softmax layer for classification of the object shape, the soil type and object's material type. Another methodology, reported in studies^41,42^, is lining detection by means of permittivity mapping of structures in the subsurface structures via specialized CNN, and deep neural network models. Also, time frequency domain and its features^43^ have been used with neural network for tunnel lining. Yet another approach employing deep neural network architectures^44^ has been used to acquire permittivity inversion of geometrical configurations of buried objects. In addition to this methodology, subsurface pipes have been detected and localized on GPR image with deep learning based back projection algorithm^45^ and subsurface targets with different shapes could be reconstructed with deep learning networks^46,47^.

Characterization of buried objects in terms of their detection and identification is only one of possible tasks considered in the literature. Other problems include estimation of characteristic features such as material shape and material type classification, object localization, estimation of dielectric properties of the subsurface medium, and classification and or quantification of the object size^24,25,32^. To solve these tasks, AI-based surrogate modelling techniques are proposed, particularly those consisting of cascaded networks^4,9,24^. In an example case study, B-scan images generated by means of gprMax tool^39,40^ are pre-processed, and windowing operation is applied on pre-processed B-scan images to extract the amplitudes most related with the object^24^. The extracted amplitudes, the outcomes of material type classification, hyperbola curvature, and the depth of the object are utilized as inputs to estimate the object dimension through Gaussian process (GP) regression^24^. A regression framework^4^ has been presented to estimate the water content of subsurface, the depth, and the radius, based on the compressed reflected signals. This methodology provides a demonstration of cascaded configuration of models, with water content and depth from the regression parameters being handled independently of each other, and the radius being dependent on the other predicted characteristics^4^. A-scans are handled as inputs for buried object characterization^4,5^ to generate 2,000 training samples thereby improving the prediction performance. Furthermore, a study of buried object characterization with the A-scan analysis^48^ and by using unprocessed sparse training data set including a vector (A-scan and number of A-scan combination) has been presented. In the mentioned works, the A-scan analysis is used as a practical processing method that can significantly reduce the computational burden of data generation^4,5,48^.

The state-of-the-art analysis presented above indicates that characterization of buried objects is a multi-aspect problem addressed using a large variety of data types and dataset configurations. On the one hand, utilization of complex data structures enables extraction of more comprehensive information about the object, in particular, its special allocation and/or estimation of its orientation and size. On the other hand, it increases the numerical challenges as well as computational costs associated with data processing and building workable representations (surrogate models). Various combinations of data structures and modeling methods offer different trade-offs with respect to the mentioned factors. Notwithstanding, the techniques that offer improved computational efficiency while being able to handle complex data structures of different types, are yet to be developed.

This work addresses the issues outlined in the previous paragraph and deficiencies of the existing surrogate-assisted buried object identification methodologies. In particular, the focus is on reducing the computational cost of creating surrogate models for object characterization and improving their reliability. In order to investigate the matter, variable data structures are taken into account for buried object characterization. These include (I) the commonly used data type of received reflected signals in the form of B-scans (2D data), (II) the extracted features from raw time signals as PCs and SFs (mean, variance, standard deviation, skewness and kurtosis), and (III) consecutive A-scans (1D data). Data generation and management affects the computational cost of data handling, in particular, constructing behavioral surrogate models, which is also a problem for surrogate models created from experimental data, as gathering of large numbers of samples is either extremely costly or even prohibitive. Furthermore, data augmentation should not be applied in association with regression approaches (as opposed to classification approaches), because each input scenario represents a mapping to a unique output. Hence, augmentation leads to unreliable prediction results. The major goal of this work is to introduce fast and accurate surrogate modeling approaches and propose a novel deep regression network (DRN) that enables a reliable prediction of characteristic parameters of the object independently from each other, and simultaneously with time frequency spectrogram (TFS) data. Furthermore, characteristic parameters are estimated without any clutter reduction method and using linearly-sampled sparse training data set. In addition to presenting a novel model with a unique-methodological approach, specifically, transforming consecutive A-scans to TFS data, the benchmarking results with the state of the art techniques from the literature are presented in terms of estimating characteristic parameters of the buried object. The object characterization process is applied independently from the dielectric features of subsurface media (the type of subsurface medium), in detail these features are not used in input and output of the surrogate models.

The main contributions of this study can be summarized as follows.

(I) The development of data diversification approaches, followed by producing new data sets from raw time signals. These offer an alternative to approaches commonly employed in the literature (image processing, hyperbola investigations on B-scan images, feature-based object characterization in classification approach);
(II) The development of data driven surrogate modeling technique for buried object characterization involving sparse linear sampling scenarios, without using any clutter reduction operations and B-scan image processing. In particular, background subtraction or removing ground reflections might be a challenging issue for buried object scenarios that involve in more than one dielectric features of dispersive subsurface media. Herein, four different subsurface media are taken in consideration to analyze the effect of complexity arising from the environments with different dielectric permittivity (not considered as a surrogate model input).
(III) The development of a novel surrogate modeling framework, DRN using a new data type of received reflected signals. The presented approach capitalizes on features extracted in the time and frequency domain in the form of TFS data transforming from consecutive A-scans instead of concatenated A-scans version (B-scan). When the proposed method DRN is used with TFS data structure, it provides computational efficiency with regards to low computational cost and high achievement.

The remaining part of the paper is organized as follows. Section “Buried object characterization task. GPR model and data structures” explains formulation of the buried object characterization task, configuration of the GPR model, description of the utilized data sets, as well as definition of the object characterization case studies considered throughout the work. Variable data structures for buried object characterization in terms of estimation performance and computational cost are also investigated in this section. Section “Data driven surrogate models”, elaborates on the proposed data driven surrogate modeling approach, specifically a DRN framework customized for TFS data. Also, customized DRN framework with TFS data is the proposed methodology for the solution of defined problem in this study. Section “Surrogate modeling for buried object characterization” discusses validation of the proposed surrogate modeling approach, as well as investigates its operation under noisy data and measurement data. Section “Conclusion” concludes the work.

## Buried object characterization task

### GPR model and data structures

This section explains the formulation of the underground object characterization task, and introduces the computational model of the data acquisition system, GPR employed as the major object identification tool. The datasets utilized for training and testing of the surrogate models for different case studies are also discussed. In addition to the contributions presented in the article, some basic information is also presented in this section so that readers from all disciplines can better understand the subject.

### Problem formulation

**Figure 1:**
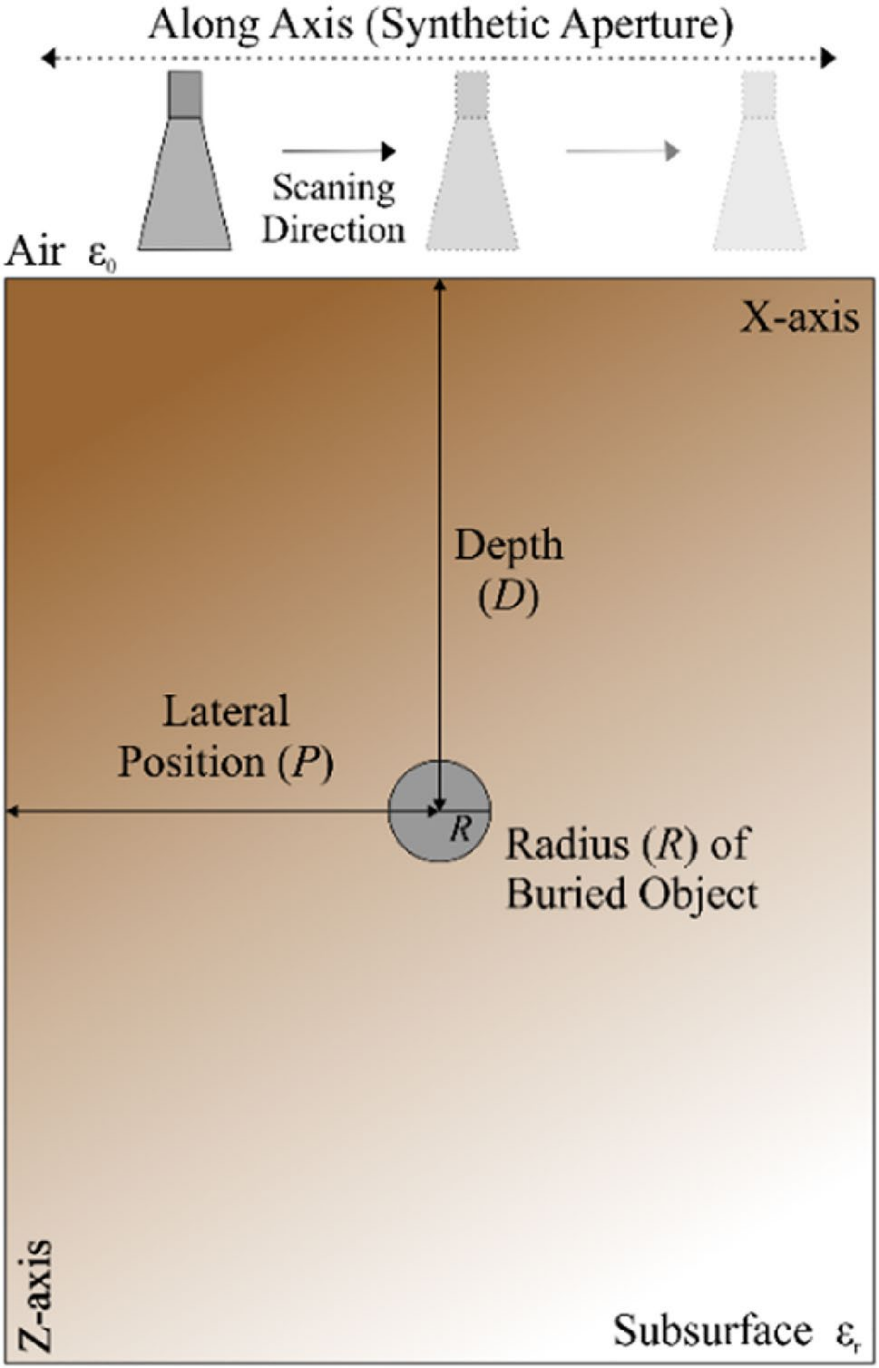
Buried object identification, explanation of terms. The parameters to be identified include *R* (object radius), *D* (object depth), and *P* (lateral position).

Herein, the problem considered is a prediction of characteristic parameters of a buried object with cylindrical shape simultaneously and independently from each other. The parameters of interest include the object radius *R*, depth *D*, and lateral position *P*. The diagram of a two-dimensional GPR model (data acquisition system) shown in Fig. 1 also explains the meaning of the parameters of the object to be characterized.

### Configuration of GPR model

#### Data acquisition scenarios

**Figure 2:**
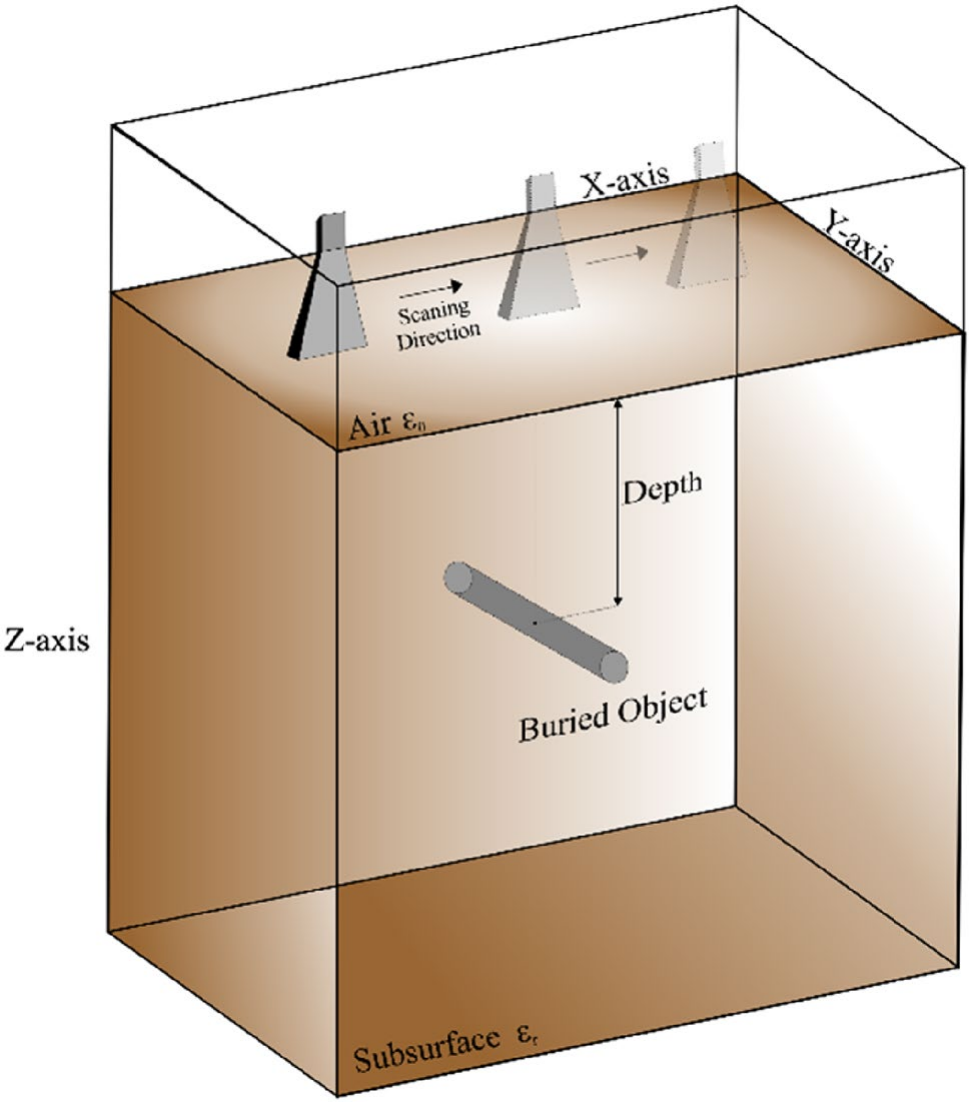
GPR model configuration in 3D-view utilized in this work to solve the buried object characterization task. Shown are the transmitting/receiving antenna, subsurface domain, and a cylindrical PEC buried object.

In this work, the object characterization problem is addressed using a GPR model. The GPR system consists of the three main components: transmitting and receiving antenna, cylindrical PEC object, and subsurface medium. For the purpose of data acquisition, the GPR model (including the buried object) is evaluated by means of 3D full wave electromagnetic (EM) analysis. A C-band pyramidal horn antenna has been used as transmitting/receiving antenna in monostatic configuration^14^. The antenna is placed in a close proximity of the air-ground surface. A Modulated-Gaussian signal (center frequency-6 GHz) is used as the transmitted signal. In this case, *t* = 12 ns. The buried object is modelled as cylindrical PEC object such as a wire, a pipe, or a rebar. The subsurface domain dimensions are defined as 400 × 300 × 500 mm. Figure 2 shows a 3D view of the data acquisition system.

The subsurface is assumed to be a dispersive subsurface medium and its dielectric properties are defined by the extended Debye model^49^ according to the percentage of water content. It is assumed as a basic one for a GPR system. However, it should be mentioned that the main contribution of the work is to propose a fast and accurate, computationally efficient data driven surrogate modeling approach with a new data structure, TFS for estimation of characteristic parameters of a buried object independently from each other, and simultaneously with different subsurface media in terms of dielectric features. The dielectric features of subsurface media can be described as

1 $$ \varepsilon = \varepsilon_{\infty } + \frac{{\varepsilon_{S} - \varepsilon_{\infty } }}{{1 + jwt_{0} }} + \frac{\sigma }{{jw\varepsilon_{0} }} $$

where* t*_0_ is the relaxation time*,* [*ε*_*s*_, *ε*_∞_] relative permittivity at zero frequency and relative permittivity at infinite frequency, whereas *σ* the soil conductivity. Four varied types of soil media are chosen in accordance with their water content of 0.2%, 2.8%, 5.5% and 6.2%^49^ via this model. The specific media parameters have been gathered in Table 1. Also, no pre-processing techniques were applied to eliminate air-ground reflections and the effects coming from the underground. This is in contrast to many approaches presented in the literature because of the usage of techniques for background subtraction or removing ground reflections, clutter reduction.

**Table 1:** Extended Debye parameters^49^ of the four types of subsurface media employed in this work. The main distinguishing parameter is water content.

Subsurface case	WC %	${\varepsilon }_{s}$	${\varepsilon }_{\infty }$	${t}_{0}$[ns]	σ [${\Omega }^{-1}{\text{m}}^{-1}$]
1	0.2	4.814	4.507	0.82	6.06⋅10^–4^
2	2.8	6.75	5.503	2.28	2.03⋅10^–3^
3	5.5	8.63	6.023	1	5.15⋅10^–3^
4	6.2	9.14	5.93	0.8	6.7⋅10^–3^

The GPR model presented above has been used to generate data, used to construct and validate the surrogate models employed for object characterization. The parameter space of object type and location scenarios are defined in a four-dimension system [depth, lateral position, radius, water content of subsurface]. The training data is allocated using a rectangular grid of the size 7 × 3 × 5 × 4 in order according to the defined four-dimensional system, with the total number of 420 sample points. Eventually, these sample points are used to generate training data set. The details can be found in Table 2. It should be emphasized that in order to ensure computational efficiency of the modeling process, a sparse dataset is used. Note that for each of 420 scenarios, sixteen A-scans are generated through EM analysis. For the proper performance evaluation of the proposed model and benchmark models, the testing data contains randomly selected 81 scenarios designated via Latin Hypercube Sampling (LHS)^50^ to prevent over-fitting of the model. The training scenarios were not used for testing the surrogate models. The testing dataset is entirely distinct from the training one in terms of the considered buried objective parameter sets.

**Table 2:** Design of the training data set for data driven surrogates solving the considered GPR problem.

Parameter	Parameter range	Step size
Depth of the buried object [mm]	100–400	50
Lateral Position of the buried the object [mm]	140–280	70
Radius of the buried object [mm]	10–50	10
Water content of the soil [%]	0.2; 2.8; 5.5; 6.2	
Total # of Scenarios	420	

### Data structures for buried object characterization

This section discusses the various data structures used in this work in the context of GPR-based object characterization. As mentioned earlier, the outcome of the GPR model are A-scans, which are time-varying normalized power amplitude signals (1D signals) obtained at a single point alongside the scanning axis (synthetic aperture). The length of the A-scan is 600 (time steps). A B-scan image is constructed by concatenating all A-scans (here, sixteen) gathered along the aperture.

In the literature, different arrangements of data structures have been used to solve the inverse GPR problems, including B-scan images (2D data), extracted features, as well as the A-scan signals, as outlined in Sect. 1. In this work, three types of data structures are considered as briefly discussed in following sections Case 1- raw 2D data (B-scans), Case 2- features extracted from raw time signals, Case 3- 1D data set of raw time signals (consecutive A-scans) and their relevance from the point of view of improving the computational efficiency and reliability of the object characterization process are analyzed. The following cases are considered: Case 1 – raw 2D time signal, Case 2 – features extracted from raw time signal, and Case 3 – raw 1D A-scan signal.

### Case 1 – raw 2D data (B-scans)

**Figure 3:**
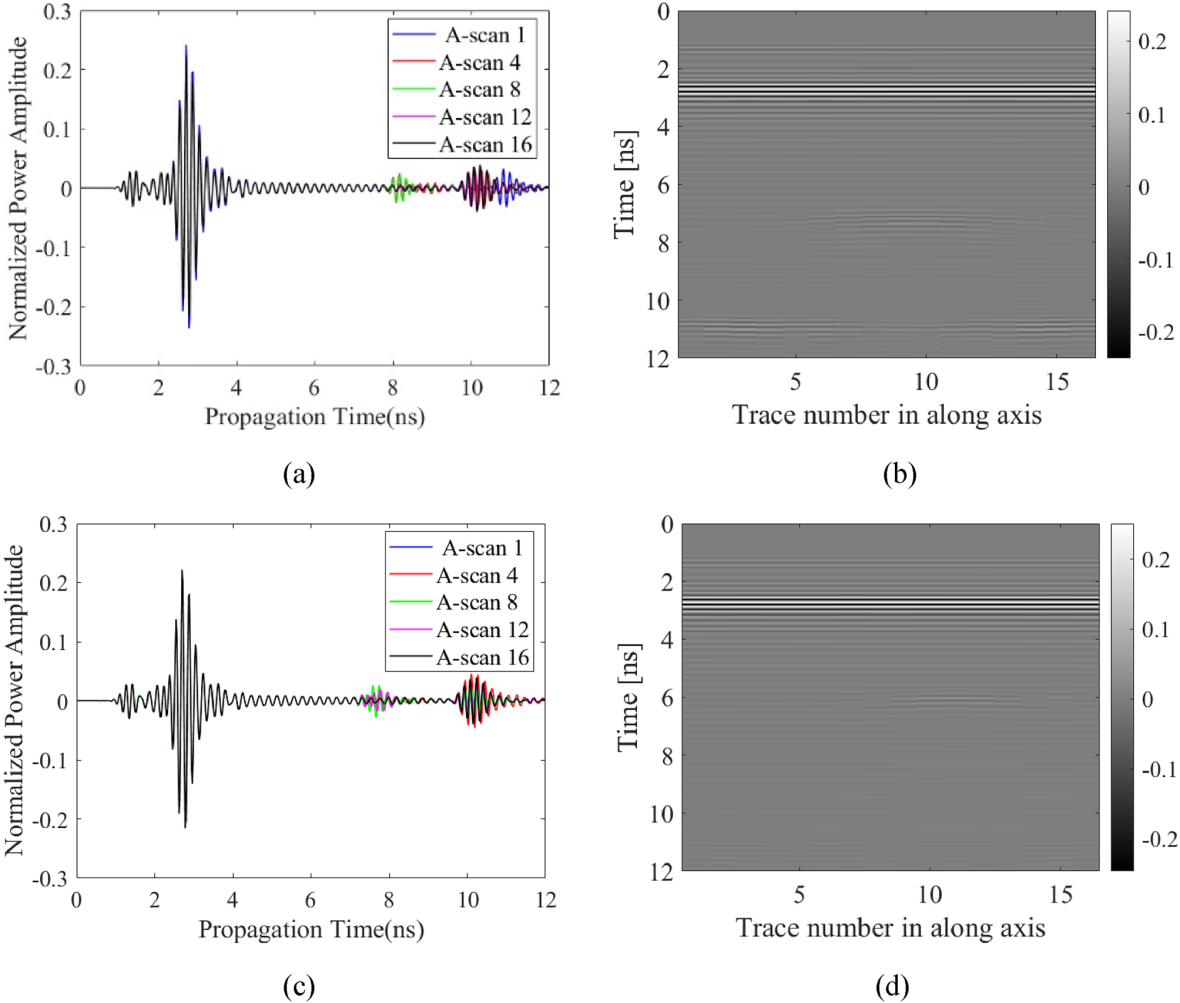
Sample raw A-scan signals and B-scan images constructed therefrom for two scenarios. The first scenario consists of radius 32 mm, depth 306 mm, lateral position 214 mm, and water content of 2.8%: (**a**) raw A-scans, (**b**) B-scan image. The second scenario consists of radius 27 mm, depth 218 mm, lateral position 244 mm, and water content of 5.5%: (**c**) raw A-scans, (**d**) B-scan image.

The primary data type considered in this work is raw B-scan data. Raw time signals in the form of 2D data are generated by combining all A-scans obtained along the synthetic aperture, as indicated in Fig. 3 for sample scenarios. The raw B-scan is represented as a matrix ***E*** = [*E*_*ij*_], with *i* = 1, …, 600, and *j* = 1, …, 16. B-scan consists of received reflected normalized power amplitudes for 600 time steps and 16 positions of the transmitting/receiving antenna at the along axis.

### Case 2 – features extracted from raw time signals

**Figure 4:**
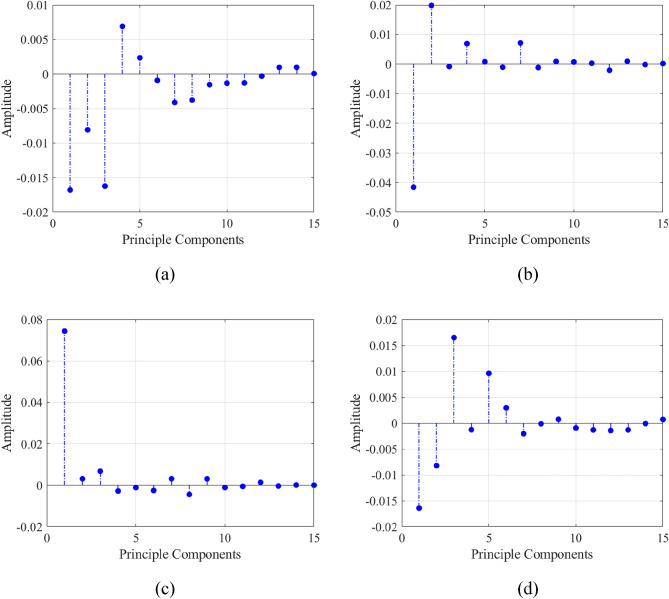
Sample principal components of A-scans (A-scan ID: 4, 8, 12 and 16) for the scenario corresponding to radius 27 mm, depth 218 mm, lateral position 244 mm, and water content of 5.5%: (**a**) A-scan ID: 4, (**b**) A-scan ID: 8, (**c**) A-scan ID: 12 and (**d**) A-scan ID: 16.

The second type of data considered in this work are features extracted from raw B-scan data. This data includes principal components (PCs) as well as selected statistical features. A part of extracted features includes PCs of B-scan, which are extracted using PCA, similarly as in studies^32,33^. As mentioned earlier, raw B-scan data has the size of 600 × 16. The array of principal components of the B-scan obtained upon applying PCA has the size of 16 × 15. The rows correspond to the subsequent A-scans, whereas the columns are the components. Figure 4 shows the example of PCs extracted from a B-scan for exemplary scenarios. Apart from the PCs, statistical features (SFs) are extracted as follows

2 $$ M = \frac{1}{N}\sum\limits_{i = 1}^{N} {PA_{i} } $$

3 $$ V = \frac{1}{N - 1}\sum\limits_{i = 1}^{N} {\left| {PA_{i} - M} \right|^{2} } $$

4 $$ STD = \sqrt {\frac{1}{N - 1}\sum\limits_{i = 1}^{N} {\left| {PA_{i} - M} \right|^{2} } } $$

5 $$ S = \frac{{E\left( {PA - M} \right)^{3} }}{{STD^{3} }} $$

6 $$ K = \frac{{E\left( {PA - M} \right)^{4} }}{{STD^{4} }} $$

where *M* is the mean, *V* is the variance, *STD* is the standard deviation, *S* is the skewness, *K* is the kurtosis. Here, *E*(*x*) stands for the expected value. The extracted features PCs and the SFs matrix are transformed to a 1D feature vector. Its size for each B-scan is 1 × 320. It should also be mentioned that other types of features can be extracted, such as the minimum and maximum. However, these give information about the reflections from air-ground boundary and are therefore irrelevant from the point of view of characteristic parameters of the object. Mean is the average value of normalized power amplitude (PA) for a single A-scan (cf. (2)), where *N* = 600 is the number of discrete-time samples (here, 12 ns). Variance (cf. (3)) is obtained as average amplitude of squared departures from the mean value. Another feature is standard deviation, cf. (4), determines the spread of the power amplitude in the time series. Skewness, cf. (5), evaluates the asymmetry of the PA with respect to the mean value. Finally, kurtosis (cf. (6)) measures the normal distribution of PA, i.e., it describes the shape of the tail histogram. It should be emphasized that the main aim is to achieve low computational cost by reducing data dimension and size. In addition, it may be possible to increase the characterization performance with the regression approach, as in the classification approach widely used in the literature. The feature-based data set consists of the most common extracted features of GPR signals used in classification studies and clutter reduction (pre-processing) techniques. Some features are also analyzed such as entropy, root mean square and singular values while preparing the manuscript, but the feature-based data set was not successful as aimed for characteristic parameter estimation. Also, it was observed that addition of features is not change the object characterization performance so much and increase the computational cost for data handling, data set processing (feature extraction), so more analysis and extended feature-based data set could not be added and the commonly used features are analyzed for solving defined problem.

### Case 3 – 1D data set of raw time signals (consecutive A-scans)

**Figure 5:**
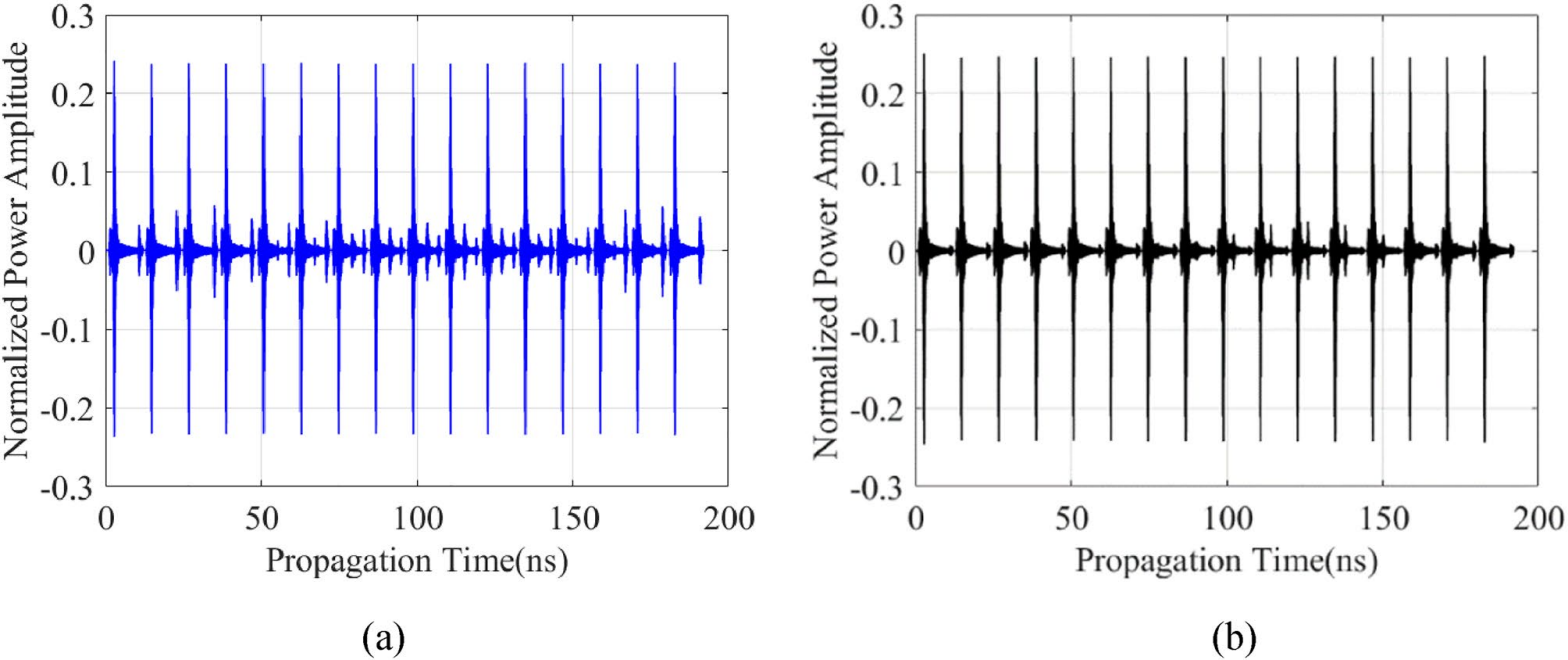
Samples of B-scans in a form of consecutive A-scans from test data set for (**a**) a scenario of radius 32 mm, depth 306 mm, lateral position 214 mm, and water content of 2.8%; (**b**) a scenario of radius 27 mm, depth 218 mm, lateral position 244 mm, and water content of 5.5%.

The last type of data structure considered here is raw B-scan data matrix converted into 1D consecutive A-scans. This operation results in a single (one dimensional) vector. Given the size of B-scans, which contain 16 pairs of A-scans of size 1 × 600 (recall that A-scan is a time-varying normalized power amplitude signal obtained at a single point along the scanning axis), the consecutive vector size is 1 × 9600 for each B-scan scenario. Figure 5 shows some examples of consecutive A-scans for specific scenarios.

### Data structures: advantages and disadvantages

As mentioned earlier, the purpose of exploring several types of data structures was to assess their relevance from the point of view of surrogate-assisted object characterization. In Case 1, the data sets are prepared by using unprocessed, raw B-scans obtained from the GPR model and used as inputs of the surrogate models. As it turns out, a construction of reliable data driven models is impeded by the sheer complexity of this type of data. In other words, although B-scans contain the most complete information about the system at hand, its handling (here, in terms of constructing data driven models) is intrinsic.

The second type of data in the form of features extracted from B-scans (Case 2) is used to reduce the data complexity, thereby facilitating its handling, also in terms of rendering accurate surrogate models. At the same time, the goal is to evaluate possible trade-offs between reliability and computational efficiency of object characterization. It should be mentioned that the literature indicates that utilization of feature-based data does lead to accuracy degradation, particularly in classification problems^8,32,34,36^. Finally, in Case 3, to obtain low error rate and improve computational efficiency, consecutive A-scans (1D raw data) are used as the input instead of concatenated A-scans. Another goal here is to compare performance of various surrogates by means of the state-of-the-art techniques. The comparison of the performances between the surrogates was not possible with the 2D raw data (raw B-scan). In this case, the information carried by the data is the same, yet only because its shape changed, an improvement has been achieved in terms of the computational cost of the training process. Nonetheless, this data type could not reach the prediction performance at the intended level. Furthermore, raw 1D time signals are handled by transforming time frequency spectrogram, TFS. The aim is to take advantages of the properties of the data in time and frequency domain, and to ensure low computational cost by changing the size of data. The proposed data handling is used by a new DRN framework as explained in detail in next section, deep regression network (DRN) customized for time frequency spectrogram (TFS) model.

## Data driven surrogate models

This section discusses the data driven surrogate models used for characterization of buried object with regards to estimate radius, depth and lateral position. In particular, the proposed DRN model is explained with the benchmark models including their configurations and hyper-parameters. In all cases, MATLAB^51^ R2022a has been used as the primary programming environment for generating data structures, as well as training and testing of the models.

### Deep regression network (DRN) customized for time frequency spectrogram (TFS) model

Herein, a new deep-learning-based framework is introduced. More specifically, a deep regression network (DRN) customized for TFS data of consecutive A-scans is developed. This model uses TFS data (2D image) obtained by using the short time frequency transform (STFT) of consecutive A-scans, 1D signals. STFT of 1D signals, a sequence of FFT of windowed of 1D signals, offers a joint distribution that enables analysis both in time and frequency domains^52^. In addition, the time frequency spectrogram (image, 2D data) has been utilized for classification of sound signals in some CNN-based, deep learning investigations^53^. The proposed data type of received reflected power amplitude signal, time frequency spectrogram with DRN framework has been constructed, partially taking inspiration from the previously mentioned developments. The proposed surrogate model may be considered as similar to the networks including DL algorithms. However, the proposed DRN network structure presents a novel model and uses a new data structure, TFS for buried object characterization in terms of estimating of the characteristic parameters in a way that is reliable and computationally efficient. Its main structure includes convolutional and fully connected layer blocks in a pyramidal configuration. Every block consists of a batch normalization (BN) layer, convolutional filter, maximum pooling layer and activation function and every fully connected layer block consists of fully connected layer and BN layer similar with the MLP configuration. Therefore, the proposed DRN network have similar features with DL and MLP but it is a new customized network model for TFS data. In the literature, there are many network structures based on deep learning, and each of them is customized, have specific hyper parameters, kernel sizes or cardinalities.

**Figure 6:**
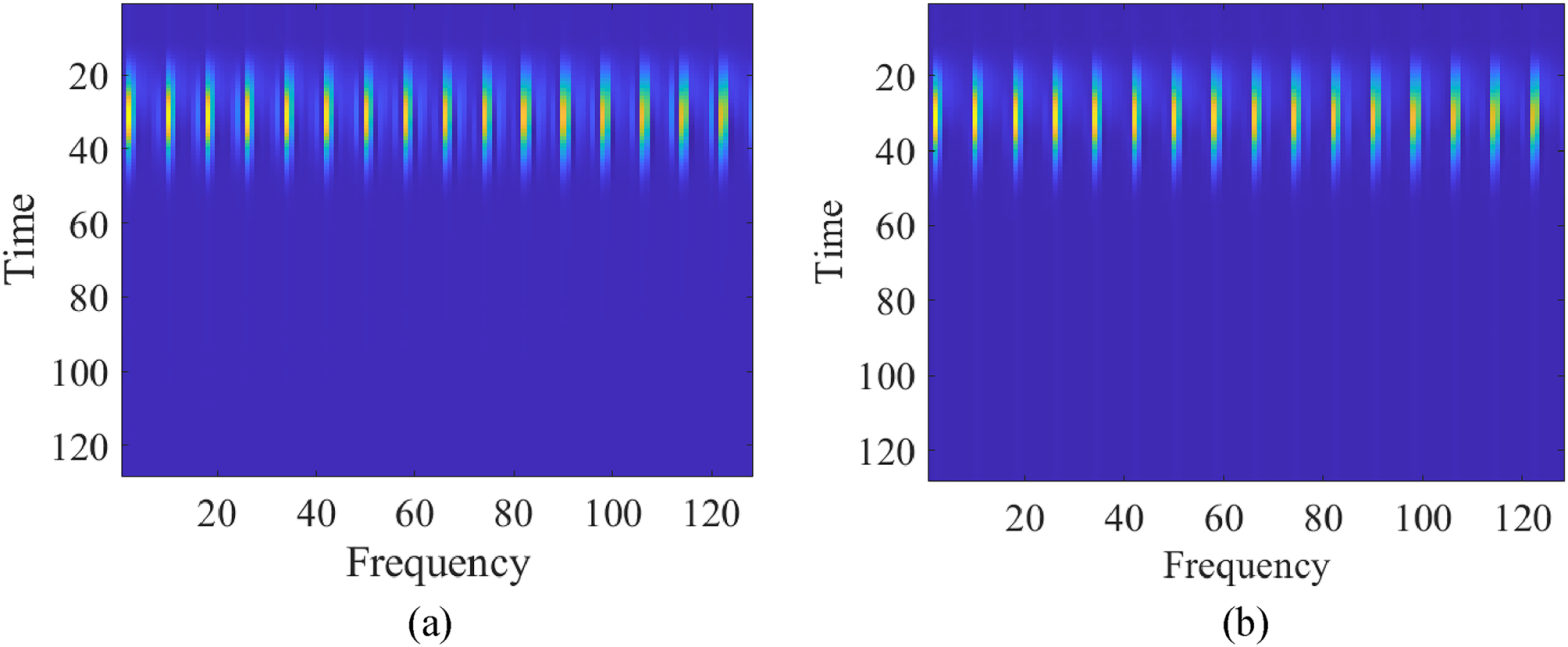
Samples of the TFS image of consecutive A-scans from the test data set for: (**a**) a scenario of radius 32 mm, depth 306 mm, lateral position 214 mm, and water content of 2.8%; (**b**) a scenario of, radius 27 mm, depth 218 mm, lateral position 244 mm, and water content of 5.5%.

The proposed framework includes a number of convolutional filter blocks and fully connected (FC) layers^54^. The consecutive A-scans dimension is 1 × 9600, and a window function is selected as 32-point (32-sample segment) Kaiser window^55^ with the shape factor of five. At the first stage, zero-average of consecutive A-scans are taken. After that, the blocks with the length of 32 are extracted from intervals which are specified by a sixteen-sample overlap between the adjoining segments. Then, the blocks are multiplied by the Kaiser window^55^, the purpose is to prohibit the spectral leakage while extracting the time frequency spectrogram. Subsequently, the length of FFT are selected as 256. Because of being symmetrical of FFT for real signals, the one-sided portion of the spectrogram is received. Complex numbers are converted to real numbers by taking their magnitude. Thus, the magnitude spectrum has been calculated to process the TFS as an image. The TFS data of consecutive A-scans are obtained and the dimension of the images are reduced to 128 × 128. Figure 6 shows the TFS images for the sample test scenarios.

**Figure 7:**
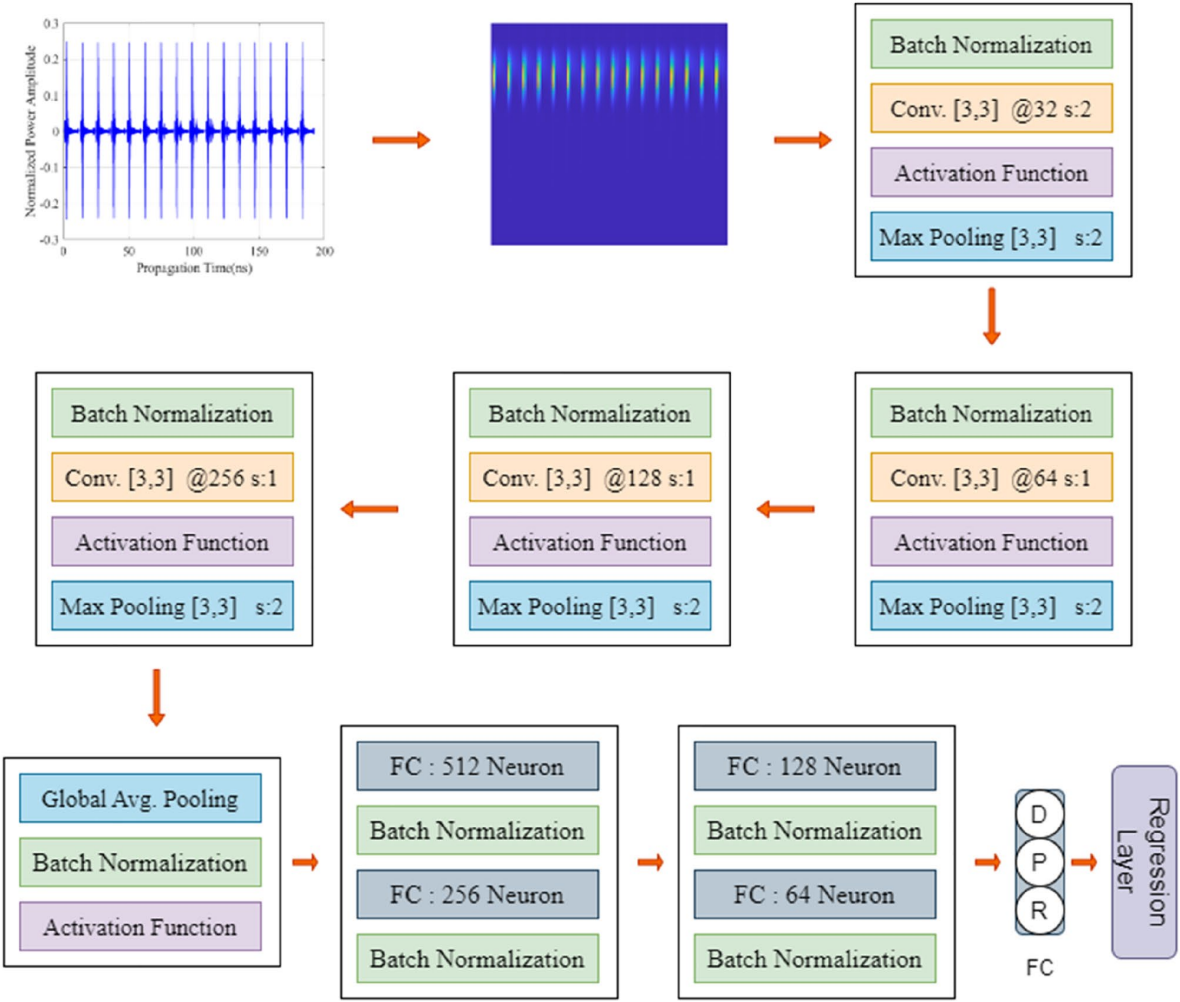
The architecture of the proposed DRN framework customized for time frequency spectrogram.

The DRN model developed to represent the data comprises of seven major blocks as demonstrated with its configuration in Fig. 7. The input layer takes the TFS data with the size of 128 × 128 × 1. After that the four major blocks including convolutional layers extract features from TFS data and down sample the extracted features. Another layers in the major blocks are batch normalization (BN)^56^ and Leaky ReLU layer which is used for activation function. These layers apply the following data processing operations to extracted features from TFS data. The last layer of the major blocks, pooling layer divides the data into pool and reduces the data maximum of each pool. In the fifth block, global average pooling layer^57^ is performed by computing average values in each dimension of input data. Thus, a feature vector with the size of 1 × 1 × 256 is derived from the TFS data. As demonstrated in Fig. 7, the numbers of filters of the convolutional layers between 32 and 256 in the last convolutional block in a pyramidal configuration. Each convolutional layer contains 3 × 3 filters and 2-strided except the first convolutional layer, it is 1-strided for feature extraction of TFS data. Also, each maximum pooling layer contains 2-strided 3 × 3 filters for reduction of extracted features in the convolutional layers. Subsequently, within the sixth block, the feature vector is processed; this block is of pyramidal shape and contains layers of the sizes from 512 neurons down to 64 neurons in the FC layer. In the last block, the input is converted to characteristic parameters of the object to be characterized (radius, depth and lateral position) through three-neuron FC layer and the regression layer.

### Benchmark models

This subsection outlines the benchmark models employed to perform comparisons with the proposed DRN with TFS surrogate methodology used for buried object characterization. The benchmark models consist of MLP^12,17,18^, GP regression^24^, CNN^3,19,42,58^, and SVRM^59^. A brief explanation of all these models are below.

### CNN (Convolutional neural network)

CNN is a deep learning-based technique^6,21,41,42,44^. Its major ability is to automate feature extraction from input by means of convolutional filters. These filters are placed in the main layers of the CNN architectures, referred to as convolutional layers. CNN model configuration consists of a variety of blocks which are used together, such as a convolutional layer, a pooling layer, a BN layer and the activation function, lastly a fully connected (FC) layer. In this work, architecture of the benchmark CNN model varies according to dimension of the input data as CNN-2D and CNN-1D. In CNN models, the configuration and the hyper-parameters are defined as: a convolutional layer (in three blocks) after the BN layer, and activation function which is selected as ReLU layer, a pooling layer involved in the last convolutional layer. The layer following the convolutional blocks is a FC layer with three neurons corresponding to the network outputs. The final layer, the regression layer, is used to estimate the outputs, specifically, radius, depth and lateral position. The remaining user-defined parameters, like the number of the filters in the convolutional layers (32, 64, 128) and the size, also the pooling layer are designated as regards literature recommendations^3^. Furthermore, the size of the filters is arranged according to dimensionality of data sets in the analyzed cases.

### MLP (Multi-layer perceptron)

The MLP^9,12,17,18,60^ model is utilized in this work with the following properties. The activation functions are used as log-sigmoid and the model is constructed with two hidden layers including 16 and 32 hidden neurons, respectively. Furthermore, the MLP model is trained by the Levenberg–Marquardt algorithm with the maximum epoch number equal to 500.

### SVRM (Support vector regression machine)

Another benchmark surrogate model is SVRM which is involved in the class of supervised statistical learning techniques^16,18,32,59^. Herein, SVRM hyper-parameters are identified using Bayesian optimization. The kernel function which is selected as a Gaussian function for Case 3 and Radial Basis Function (RBF) for Case 2 is one of the important components SVRM.

### GP regression (Gaussian process regression)

The last model for benchmarking is Gaussian process (GP) regression. The main working principle of GP regression technique is generalizing Gaussian-Probability-Distributions to functions^24,61^. For this analysis, the selected kernel function is “matern 3/2”^62^ for numerical experiments, and the block coordinate descent method is used for estimation with the block size of 200. The selection of hyper-parameters is a significant part of AI based data driven surrogate modelling. Herein, Bayesian-Optimization is applied to obtain optimum hyper-parameters. The K-fold technique with K = 10 is used for validation.

## Surrogate modeling for buried object characterization

### Experimental results

This section discusses the results of verification experiments using the surrogates (the proposed DRN with TFS and benchmark methods). The performance of predicting buried object parameters is measured using Mean Absolute Error (MAE) expressed in millimeters, and Relative Mean Error (RME) expressed in percent. Both error metrics are described as follows

7 $$ MAE = \frac{1}{N} \times \sum\limits_{i = 1}^{N} {\left| {T_{i} - P_{i} } \right|} $$

8 $$ RME = \frac{1}{N} \times \sum\limits_{i = 1}^{N} {\frac{{\left| {T_{i} - P_{i} } \right|}}{{\left| {T_{i} } \right|}}} $$

*N*: the total # of samples, *T*_i_: Target, *P*_i_: model prediction, for the *i*th sample of test data set.

**Figure 8:**
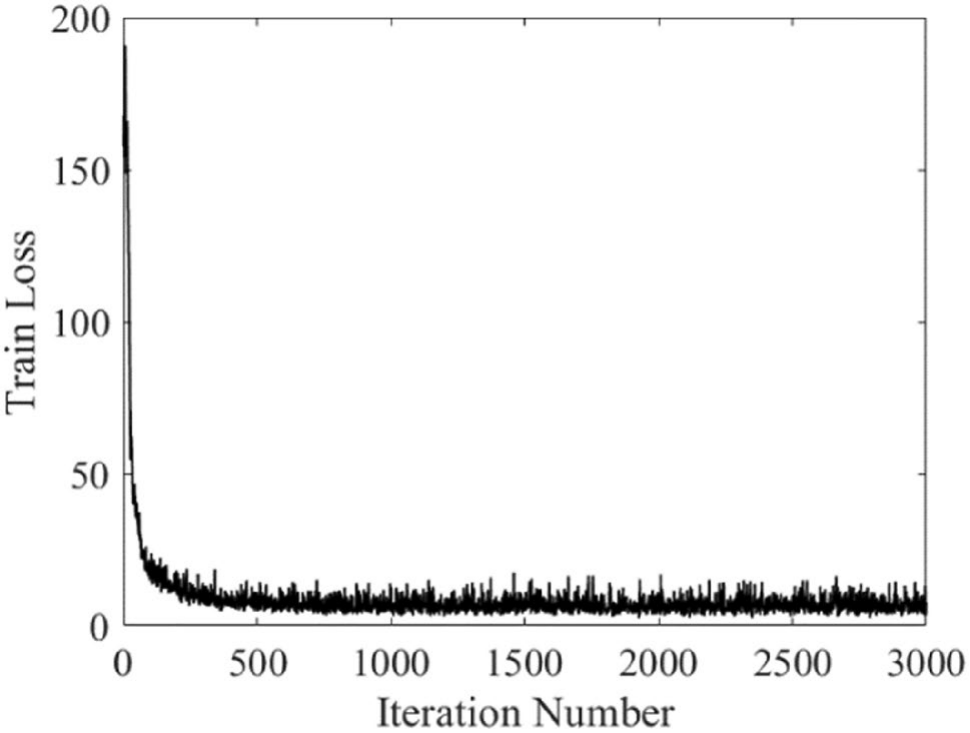
The train loss of DRN model customized for TFS data versus iteration number.

**Figure 9:**
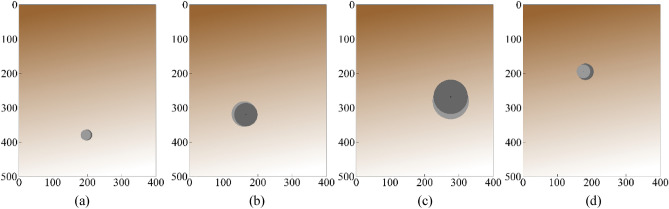
The geometrical configurations of the characteristic parameters belonging the target and predicted by DRN with TFS (the proposed surrogate model). The surrogate-predicted and the target objects indicated via the light- and dark-grey shade, respectively. The following geometrical configurations have been shown: (**a**) *R* = 16 mm*, D* = 379 mm, and *P* = 198 mm, (**b**) *R* = 34 mm*, D* = 320 mm, and *P* = 165 mm, (**c**) *R* = 50 mm*, D* = 268 mm, and *P* = 276 mm, (**d**) *R* = 24 mm*, D* = 195 mm, and *P* = 182 mm.

Table 3 represents the statistical results obtained for ten independent runs of each dataset cases and each surrogate model. Apart from the aforementioned error metrics, the training times and the required processing time for the test data are also shown. The analysis of the results allows us to formulate a number of observations. As it can be noted, the processing time for Case 1 (2D data) is the highest. At the same time, it ensures reasonably good predictive power. Extracting and processing features, associated with dimensionality reduction leads to a decrease of the model training time as observed for Case 2. However, the prediction performance is compromised as compared to Case 1. The employment of the sequential form of A-scans in characterization analysis is associated with high computational cost for some of the benchmark techniques, which is similar to that for Case 1, whereas prediction performance is insufficient. At the same time, the results demonstrate that estimation of characteristic parameters of the buried object with TFS image of consecutive A-scans using the proposed DRN framework is the best option, which allows for maintaining satisfactory computational efficiency as well as improved prediction performance (low error metrics) as compared to other types of data sets. Figure 8 demonstrates the training progress of proposed framework by showing the loss values versus the (training) iteration number. Furthermore, Table 4 demonstrates the error metrics obtained for individual characteristic parameters, computed for Case 3 analysis and the best performance-wise run out of the ten independent runs performed. Moreover, Fig. 9 represents the geometrical configurations of selected example test scenarios in terms of compatibility among the estimated characteristic parameters by using the proposed DRN with TFS surrogate model and target values.

**Table 3:** Prediction performance and computational costs for all considered surrogate models. Shown are averaged performance figures and the corresponding standard deviations computed for 10 different runs.

Case	Model	MAE [mm]	RME [%]	Training-time [min]	Evaluation duration of a single input data [ms]
Case 1 (2D data, B-scans)	CNN-2D	12.7 ± 0.8	13.6 ± 1.0	229.0 ± 5.0	18.9 ± 1.2
Case 2 (Extracted Features)	CNN-2D	17.8 ± 0.9	16.3 ± 0.9	9.0 ± 1.1	7.4 ± 2.2
	CNN-1D	21.2 ± 0.7	19.0 ± 1.3	9.0 ± 1.0	6.5 ± 1.3
	MLP	37.6 ± 2.5	33.8 ± 2.9	29.4 ± 1.2	1.7 ± 0.7
	SVRM	36.8 ± 1.8	43.7 ± 3.5	0.6 ± 0.1	0.4 ± 0.1
	GP regression	31.4 ± 0.9	31.3 ± 3.4	1.5 ± 0.1	0.3 ± 0.1
Case 3 (1D data, Consecutive A-scans)	DRN with TFS [This work]	3.6 ± 0.2	4.7 ± 0.6	18.3 ± 0.7	7.7 ± 0.6
	CNN-1D	11.8 ± 0.5	11.6 ± 0.6	187.6 ± 6.1	17.6 ± 2.7
	MLP	43.1 ± 3.4	31.4 ± 1.5	360.5 ± 9.1	1.7 ± 0.4
	SVRM	26.1 ± 1.1	24.2 ± 0.8	9.4 ± 0.6	3.5 ± 0.3
	GP regression	22.9 ± 0.1	21.6 ± 0.2	6.1 ± 0.6	1.1 ± 0.1

**Table 4:** Performance benchmarking of the proposed surrogate and counterpart models for the targeted characteristic parameters belong to the Case 3 (data shown for the best out of ten independent runs).

Model	Characteristic	MAE [mm]	RME [%]	Average MAE [mm]	Average RME [%]
DRN with TFS [This work]	Depth	4.2	2.0	3.1	3.9
	Lateral position	2.8	1.4		
	Radius	2.2	8.3		
CNN-1D	Depth	14.7	6.6	11.4	11.3
	Lateral position	14.9	7.2		
	Radius	4.7	20.1		
MLP	Depth	47.4	15.7	22.9	21.0
	Lateral position	14.1	23.4		
	Radius	7.1	32.6		
SVRM	Depth	52.5	26.6	25.1	23.4
	Lateral position	13.8	6.7		
	Radius	8.9	36.8		
GP regression	Depth	47.3	23.4	22.8	21.4
	Lateral position	13.9	6.8		
	Radius	7.3	34.1

As mentioned earlier and indicated by the results in Table 3, the best results are obtained for Case 3 (consecutive A-scans). Consequently, further performance analysis is carried out in details for this case only. More specifically, individual error metrics are investigated for all characteristic parameters, as well as the compare predicted characteristic parameters with their target values. The error values for the DRN framework are 3.1 [mm] (MAE) and 3.9% (RME), which is the average for the three considered characteristic parameters (radius, depth and lateral position). The best benchmark model, CNN-1D, demonstrates considerably worse prediction performance with the average error values of 11.4 mm (MAE) and 11.3% (RME), which is almost three times as high.

More detailed information concerning the experimental setup is provided. For Case 3 (1 × 9600 1D consecutive A-scans) the MLP model could not be constructed with the used hardware configuration due to insufficient RAM capacity. Consequently, down sampling was applied at 10-percent rate, so that the data was re-scaled to the size of 1 × 960. For SVRM (Case 3), the determined hyper-parameters of Gaussian kernel functions in optimal follow as: Kernel scale of 0.5527, Box-constraint of 981.36, and Epsilon 1.8628. The proposed DRN framework employs the Adam optimizer^63^, which is a backpropagation algorithm, for reduction of the computational cost of the deep learning-based surrogate model training^64^. In this model, the maximum epoch number is selected as 500 and the batch size is used as 64. In addition, shuffling is applied to TFS data in the proposed DRN in every epoch. While training of the CNN model, the same batch size and Adam optimizer^64^ is applied as those used for the DRN model in order to make an accurate and fair comparison. Furthermore, the learning rate is set to value of 10^–3^ until the selected epoch number reached (here, 500).

**Figure 10:**
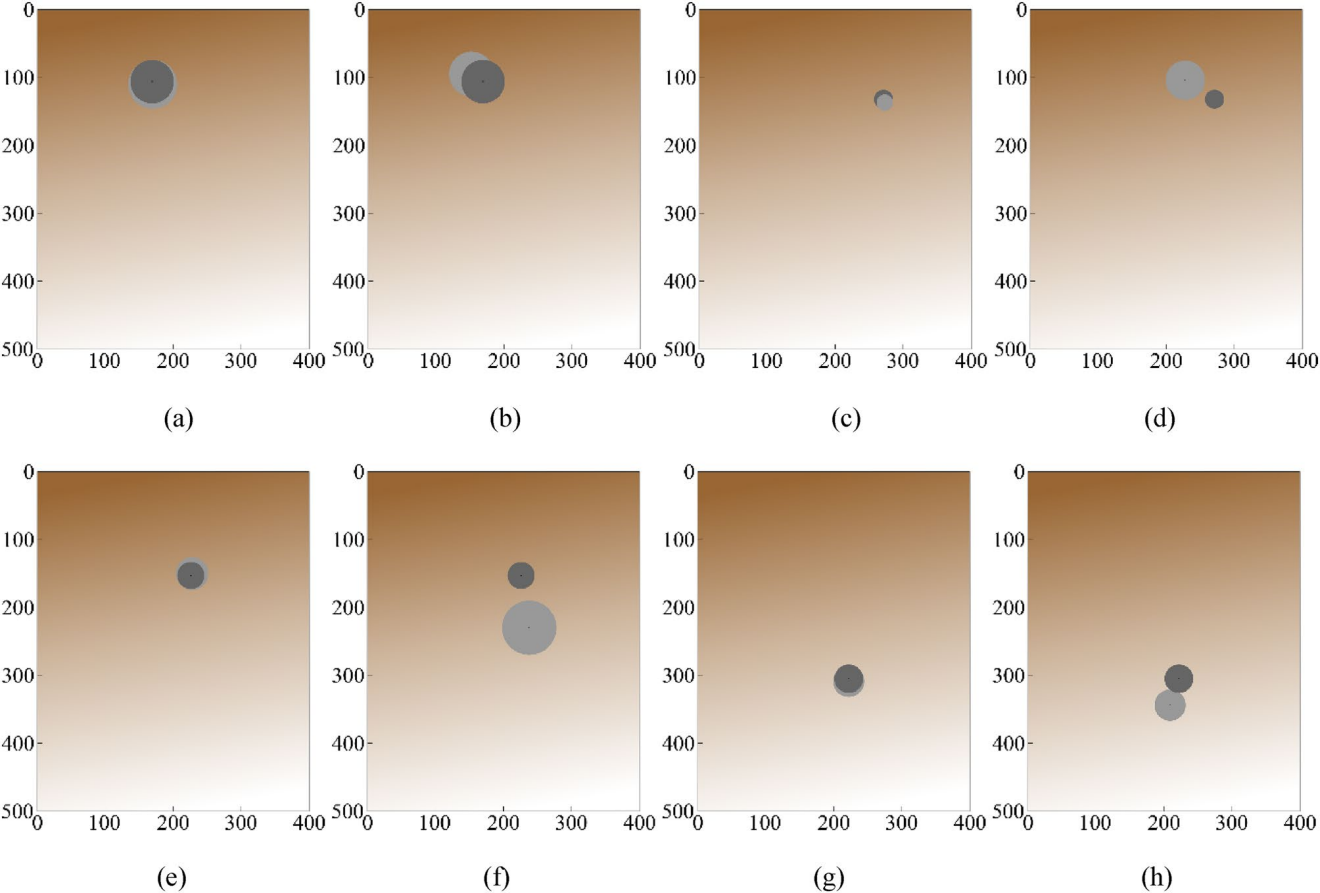
The geometrical configurations of the characteristic parameters belonging the targets and predicted by DRN with TFS (the proposed surrogate model) with the comparison network models. The surrogate-predicted and the target objects indicated via the light- and dark-grey shade, respectively: The first scenario: [34, 193, 110], (**a**) DRN with TFS, (**b**) CNN-1D; The second scenario: [21, 361, 189], (**c**) DRN with TFS, (**d**) MLP; The third scenario: [26, 244, 299], (**e**) DRN with TFS, (**f**) SVRM; The fourth scenario: [34, 193, 110], (**g**) DRN with TFS, (**h**) GP regression. All the predicted parameters in [mm] in order of [*R, D, P*].

The error metrics reported in Table 4 indicate that the proposed DRN surrogate model working with TFS data outperforms all benchmark methods by a large margin. Furthermore, the predictions concerning characteristic parameters of buried object made by the proposed surrogate are very much satisfactory. Table 5 provides another demonstration of the model performance, in the form of a comparison between actual object parameters and predictions yielded by the proposed and benchmark models, for selected scenarios. Again, the predictions of the presented DRN framework with customized TFS data are significantly closer to the true values than those obtained using other methods, for all considered cases. In Fig. 10, geometrical configurations of the actual and predicted characteristic parameters are represented for selected test scenarios in the form of a comparison between the proposed DRN framework (a version using TFS data of consecutive A-scans) and the benchmark models with consecutive A-scans (Case 3). As it can be observed, the proposed DRN surrogate with TFS yields consistent results with close proximity among the actual and the predicted and location/size of the object. This corroborates the relevance of the proposed approach to buried object characterization. Furthermore, the results obtained for the benchmark studies reported in the literature^4,6,7,24,37,48^ have been compared to the approach proposed in this study, and reported in Table 6. The compared methodologies have been mentioned in the introduction section. The table contains statistical results, the samples in training data sets and the dimensions of scanning subsurface domain corresponding to compared studies.

**Table 5:** Predicted characteristic parameters versus actual values for the proposed DRN surrogate customized for TFS and the comparison methods for some test scenarios.

Scenario ID	Model	*D*	*P*	*R*	Error (*D, P, R*) [mm]
1	True value	155	224	38	–
	DRN with TFS [This work]	146	224	40	9, 0, − 2
	CNN− 1D	176	260	39	− 21, − 36, − 1
	MLP	309	238	34	− 154, − 14, 4
	SVRM	180	228	46	− 25, − 4, − 8
	GP regression	184	220	39	− 29, 4, − 1
2	True Value	110	178	32	–
	DRN with TFS [This work]	113	175	30	− 3, 3, 2
	CNN− 1D	105	147	26	5, 31, 6
	MLP	103	229	29	7, − 51, 3
	SVRM	164	187	40	− 54, − 9, − 8
	GP regression	159	198	38	− 49, − 20, − 6
3	True Value	211	225	15	–
	DRN with TFS [This work]	209	232	17	2, − 7, − 2
	CNN− 1D	234	217	25	− 23, 8, − 10
	MLP	367	185	29	− 156, 40, − 14
	SVRM	363	209	21	− 152, 16, − 6
	GP regression	339	210	30	− 128, 15, − 15

**Table 6:** Comparison between the proposed DRN surrogate customized for TFS and the counterpart methodologies.

Methodology	Subsurface dimension	Training data set	Depth	Lateral position	Size
Ref^4^	0.5 × 0.3 × 0.4 [m]	2000 [80%]	maximum error ± 2 cm	–	radius, ± 6 mm accuracy
Ref^6^	1.0 × 0.2 [m]	2370	error < 1.5 mm and relative error < 5%	error < 7 mm	–
Ref^7^	50 × 15 [cm]	1400	–	–	accuracy 99.5%, classification of 9 different diameters
Ref^24^	4 × 5 [m]	100	–	–	size estimation, MAE 4.8 cm, Relative MAE 6.1%
Ref^37^	0.4 × 0.6 [m]	500	MAE 14.4 mm, RME 4.4%	MAE 9.0 mm, RME 6.4%	radius, MAE 2.6 mm, RME 10.8%
Ref^48^	0.4 × 0.3 × 0.5 [m]	315	MAE 10.4 mm, RME 4.7%	MAE 17.7 mm, RME 8.7%	radius, MAE 7.7 mm, RME 34.5%
[This work]	0.4 × 0.3 × 0.5 [m]	420	MAE 4.2 mm, RME 2.0%	MAE 2.8 mm, RME 1.4%	radius, MAE 2.2 mm, RME 8.3%

### Deep regression network model for time frequency spectrogram using noisy data

This section discusses further verification of the proposed data structure and surrogate model using a new noisy data set, which is generated by adding random noise^7,22,31,42,65–68^ to consecutive A-scans. The purpose is to analyze the internal and environmental noise effects of the GPR system. In the literature, different purposes have been associated with noise incorporation, such as being closer to realistic scenarios^21,22,41,58,65^, data augmentation^7,21,41,65^, as well as testing stability and sensitivity of the studied models^31,42,66–68^.

The characteristic parameters are estimated using noisy data sets generated by randomly addition white Gaussian noise^42^. This way of incorporating noise is commonly employed in the literature. For instance, random Gaussian noise have been utilized to replicate field scenarios in the context of deep convolutional network modeling for coastal hazard mitigation^65^. In a study^67^, a noise suppression method based on white Gaussian noise for GPR data had been presented, which is based on ensemble empirical mode decomposition (EEMD) approach. It should also be noted that the interior system noise of the GPR systems leads interferences with the received reflected signals, and it can be assumed to be similar to the white Gaussian noise^66^. In addition to these studies, noisy data have been used verifying stability of the model and testing the sensitivity to noise by using generated signals with different amplitudes of white Gaussian noise addition^31,42^.

**Figure 11:**
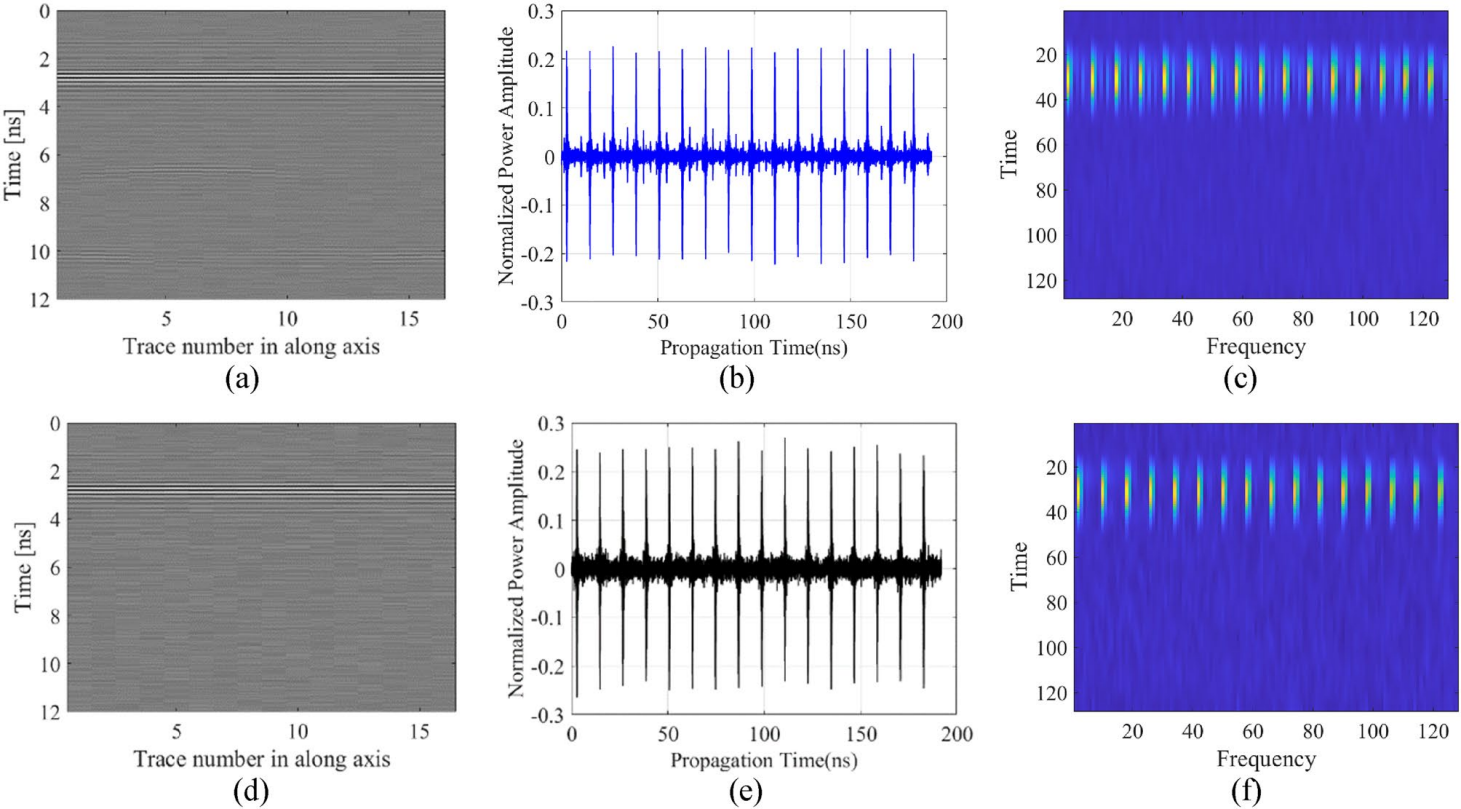
B-scan images, their consecutive A-scans and TFS images constructed for two noisy scenarios. The first scenario belongs to SNR value of 30 dB, radius 49 mm, depth 311 mm, lateral position 141 mm, and water content of 0.2%: (**a**) B-scan image, (**b**) consecutive A-scans, (**c**) TFS image. The second scenario belongs to SNR value of 20 dB, radius 15 mm, depth 211 mm, lateral position 225 mm, and water content of 6.2%: (**d**) B-scan image, (**e**) consecutive A-scans, (**f**) TFS image.

The problem defined in this verification study is solved by using 20 dB and 30 dB signal-to-noise ratio (SNR) rates white Gaussian noisy data sets to obtain conditions, which are closer to real-world and on site applications^42^. In Fig. 11, noisy data have been represented with different scenarios and two SNR rates of 20 dB and 30 dB. The noisy data sets are generated for Case 3. For the purpose of comparison, the proposed DRN framework with TFS data of consecutive A-scans is juxtaposed against the best benchmark model CNN-1D. The results obtained for both models have been presented in Table 7. In addition, the surrogate-predicted characteristic parameters have been compared to true parameters for selected test scenarios in Table 8. The reported error metrics indicate that performance of the proposed framework DRN with TFS data (average MAE of 10.1 for SNR of 30 dB and 15.8 for SNR of 20 dB) is clearly better than the best benchmark model, CNN-1D (average MAE of 20.7.1 for SNR of 30 dB and 25.2 for SNR of 20 dB). In other words, even though increasing the data complexity by adding noise has a negative impact on the overall performance of the models, the proposed approach is still the superior to the second-best surrogate CNN-1D.

**Table 7:** Prediction performance of proposed DRN with TFS and CNN-1D models for the characteristic parameters through noisy data sets.

Model	Characteristic	SNR = 30 dB			SNR = 20 dB		
		MAE [mm]	RME [%]	Average MAE [mm]	MAE [mm]	RME [%]	Average MAE [mm]
DRN with TFS [This work]	Depth	13.1	5.4	10.1	21.6	9.4	15.8
	Lateral position	10.5	5.3		15.2	7.7	
	Radius	6.5	26.0		6.8	30.1	
CNN-1D	Depth	31.5	12.4	20.7	40.7	16.4	25.2
	Lateral position	23.6	10.7		27.6	12.6	
	Radius	6.9	29.5		7.4	32.0

**Table 8:** Predicted characteristic parameters of the DRN and CNN-1D models in comparison with their actual values for some noisy test scenarios.

Scenario ID	Model	*D*	*P*	*R*	Error (*D, P, R*) [mm]
1	True value	240	150	14	–
	DRN with TFS [20 dB] [This work]	247	158	18	− 7, − 8, − 4
	CNN− 1D [20 dB]	255	169	16	− 15, − 19, − 2
2	True value	160	232	45	–
	DRN with TFS [30 dB] [This work]	167	232	42	− 7, 0, 3
	CNN− 1D [30 dB]	130	215	33	30, 17, 12
3	True value	276	201	21	–
	DRN with TFS [20 dB] [This work]	281	216	24	− 5, − 15, − 3
	CNN− 1D [20 dB]	263	178	27	13, 23, − 6
4	True value	128	160	19	–
	DRN with TFS [30 dB][This work]	132	164	25	− 4, − 4, − 6
	CNN− 1D [30 dB]	171	145	30	− 43, 15, − 11

### Analysis of deep regression network model with time frequency spectrogram using measurement data

This section presents a supplementary validation of the proposed modeling approach, DRN with TFS on the subject of performance for estimating characteristic parameters, by means of experimental data. The data has been gathered via the measurements in a “sand pool” environment, in the laboratory at Yıldız Technical University. Herein, it should be mentioned that collecting the experimental data is extremely laborious because of substantial manual efforts related to digging and accurately burying the targets, as well as adjustments/maintenance of the system. These obstacles are also the major causes for the use of data driven surrogate methodologies as primary tools in the context of buried object detection/characterization with GPR systems.

**Figure 12:**
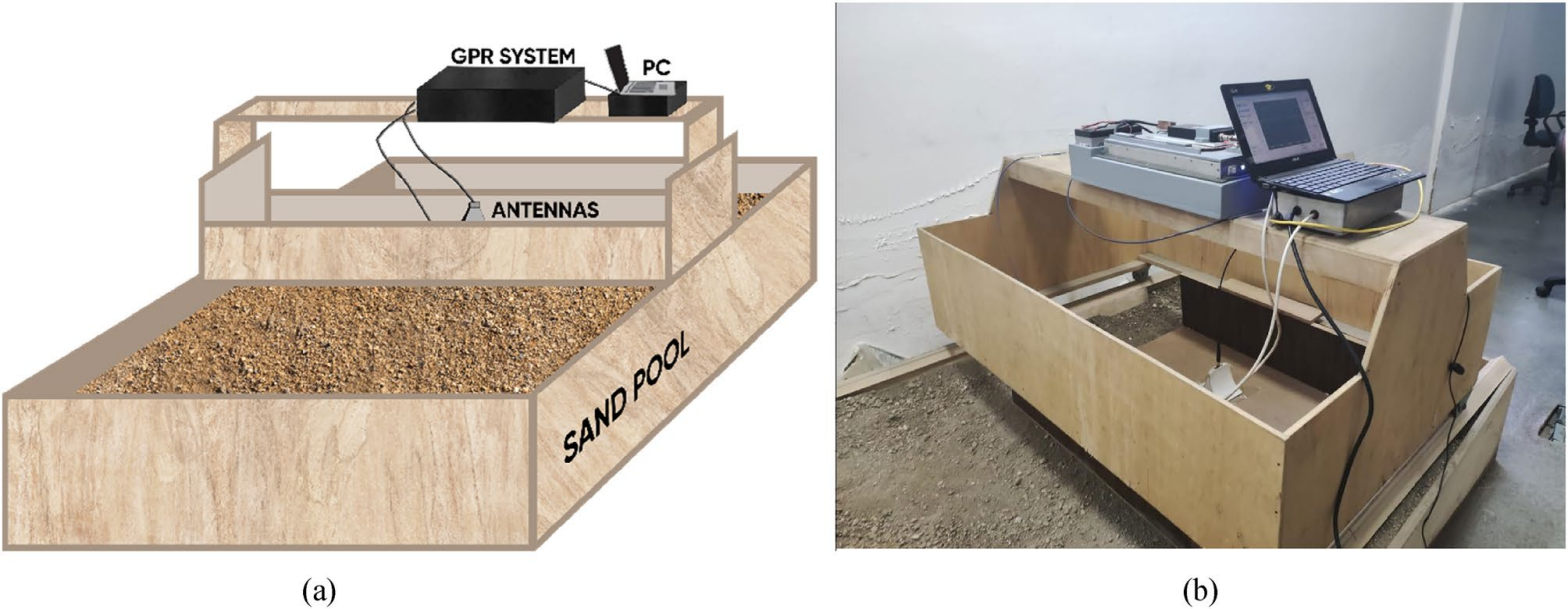
(**a**) Configuration of the measurement setup and (**b**) the picture of the measurement environment utilized to generate experimental data set for the use of proposed customized deep learning based surrogate model.

**Figure 13:**
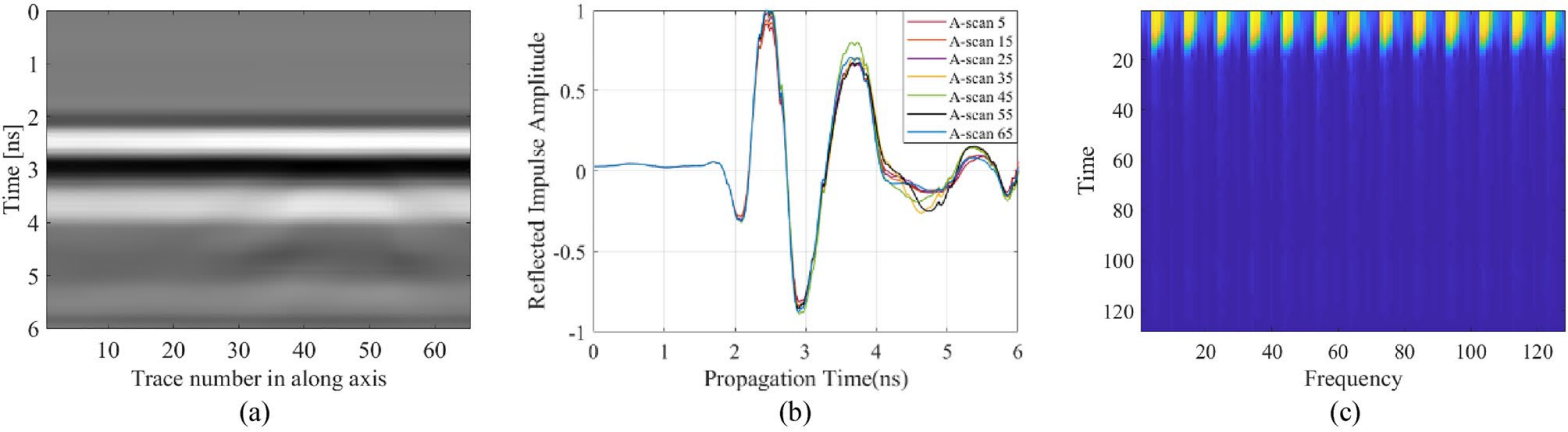
The B-scan image along with the sample A-scans, and the TFS image constructed for a selected test scenario from the measurement data set. The scenario corresponds to radius of 20 mm, depth of 110 mm, and lateral position of 770 mm: (**a**) B-scan image, (**b**) sample A-scans, (**c**) TFS image.

The main purpose here is to show that the proposed modeling approach is also feasible when used with the experimental measurements as a source of data. The B-scan data according to several scenarios are gathered by the impulse ground penetrating radar system, which is used in subsurface imaging operations^69–72^. Figure 12 demonstrates the measurement setup. Cylindrical PEC objects of different radii are buried in the subsurface. The experimental data are taken from inhomogeneous dry soil including a mixture of sand and small stones in a wooden pool, used as the scanning subsurface domain. The pool dimensions are almost 0.22 m (depth), 1.15 m (width), and 1.40 m (length). The length of the scanning path is approximately 1.40 m. B-scan data of selected scenarios is collected while the measurement setup (GPR, transmitter and receiver antennas) is manually taken steps along the scanning path above the subsurface. Each B-scan data (382 × 65) consists of concatenated 65 received reflected (time-varying amplitude) signals, A-scans with the length of 382 (discrete time step). In advance of applying the proposed methodology to the measurement data for characterizing the buried object, raw B-scan data matrix is converted into 1D consecutive A-scans (Case 3). The consecutive vector size is 1 × 24,830 for each B-scan scenario. Figure 13 shows a B-scan image, a sample of A-scans, and the TFS image of the selected scenario. The proposed novel framework utilized TFS image transformed from consecutive A-scans instead of concatenated form of A-scans (B-scan) as input, the size of which is 128 × 128 × 1. The outputs of the surrogates are the predicted characteristic parameters, i.e., radius, depth and lateral position. The measurement data set which is used to train and test the presented surrogate methodology and the best comparison model (CNN-1D) consists of 33 scenarios. The object which is buried at different positions including depth and lateral positions (such as 550 mm, 650 mm, 770 mm and 900 mm) has various radii (such as 10 mm, 15 mm, 20 mm, 25 mm and 30 mm).

**Figure 14:**
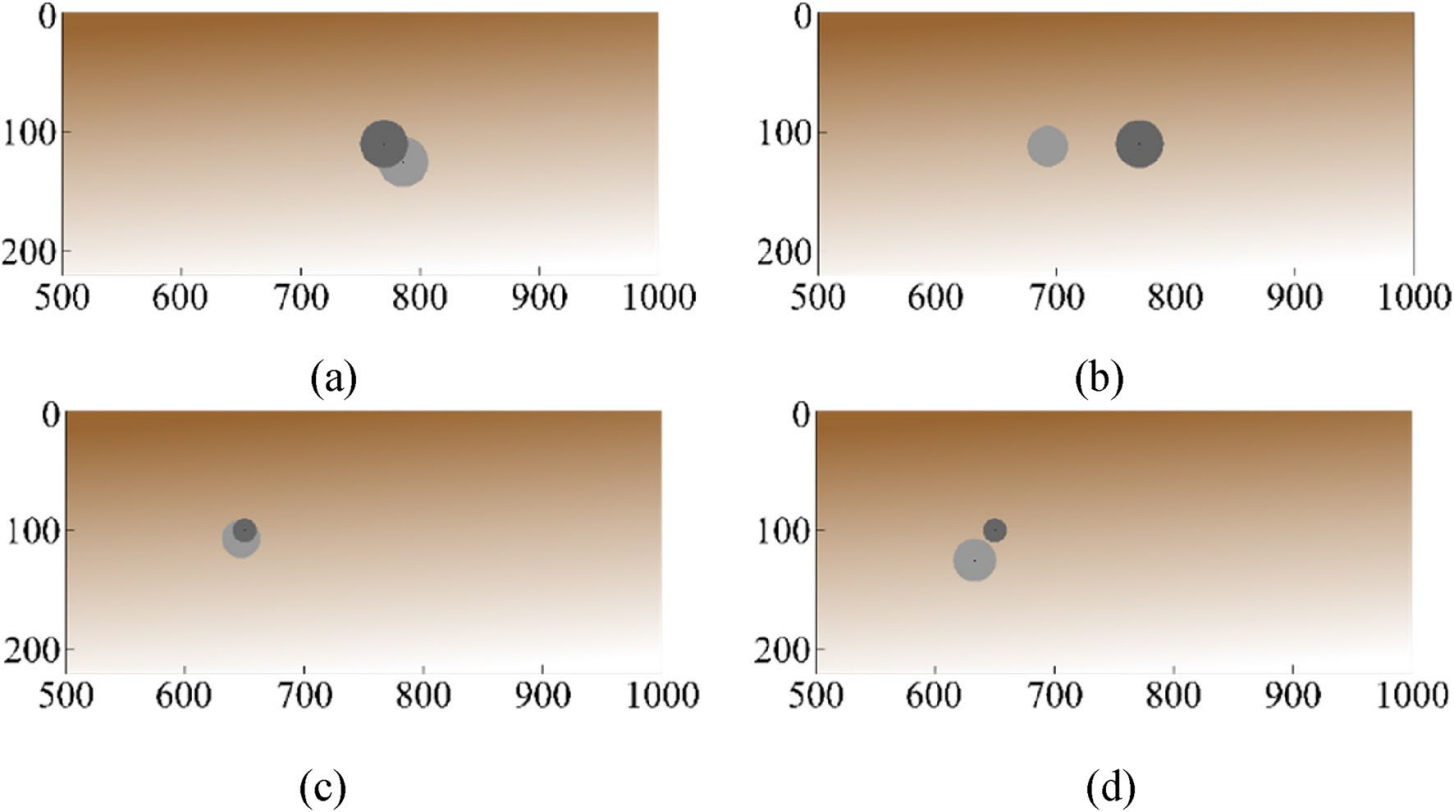
The geometrical configurations of the characteristic parameters belonging the targets and predicted by DRN with TFS (the proposed surrogate model) with the benchmark model. Models constructed with the measured data. The surrogate-predicted and the target objects indicated via the light- and dark-grey shade, respectively: The first scenario: *R* = 20 mm, *D* = 110 mm, and *P* = 770 mm, (**a**) DRN with TFS, (**b**) CNN-1D; The second scenario: *R* = 10 mm*, D* = 100 mm, and *P* = 650 mm, (**c**) DRN with TFS, (**d**) CNN-1D.

The object characterization problem considered in this validation study is solved using the measurement data. Tables 9 and 10 show the errors of the proposed model and the best benchmark technique, CNN-1D, whereas Table 11 provides numerical comparisons between the model-predicted and the actual values of the characteristic parameters. Meanwhile, Fig. 14 shows geometrical configurations of the selected test scenarios, i.e., the predicted versus actual object allocation and size. In Table 9, the average errors of determining all characteristic parameters (depth, lateral position, radius) are obtained by taking the results of various runs of the models. According to the best prediction of the models (cf. Table 10), the performance of the proposed DRN framework customized for TFS data (average MAE of 29.6 mm) is clearly preferable with respect to object characterization quality than that of the comparison model, CNN-1D (average MAE of 37.2 mm). Also, the computational cost of CNN-1D is much higher than that of the proposed model. Figure 14 illustrates the alignment between surrogate-predicted and true characteristic parameters via the geometrical configurations corresponding to the selected scenarios. As it can be observed from the geometrical configurations, visual agreement between the target and surrogate-predicted object size and position (depth and lateral position) is superior to the proposed model as compared to CNN-1D. Table 11 represents comparisons between the true and surrogate-predicted characteristic parameters with error values for sample test scenarios. In particular, the estimation of the characteristic parameters achieved via the DRN model with TFS data is satisfactory for practical purposes, as demonstrated in Table 11 and Fig. 14, and the proposed modeling approach is considerably more successful than the benchmark model, CNN-1D.

**Table 9:** Prediction performance and computational costs for proposed model, DRN with TFS and benchmark model, CNN-1D. Shown are averaged performance figures and the corresponding standard deviations computed for 10 different runs for the measurement data.

Model	MAE [mm]	RME [%]	Training time [min]	Processing time for a single input test data [ms]
DRN with TFS [This work]	32.6 ± 2.3	21.0 ± 0.9	3.1 ± 0.2	30.0 ± 4.0
CNN-1D	42.0 ± 4.2	23.6 ± 0.8	28.2 ± 1.1	79.0 ± 16.0

**Table 10:** The quality of predicting characteristic parameters using the measurement data. The table shows the performance figures for the proposed surrogate and the benchmark model (for the best out of ten independent runs).

Model	Characteristic	MAE [mm]	RME [%]	Average MAE [mm]	Average RME [%]
DRN with TFS [This work]	Depth	14.2	9.7	29.6	19.2
	Lateral position	68.7	9.1		
	Radius	5.7	38.7		
CNN-1D	Depth	23.9	16.3	37.2	22.7
	Lateral position	81.0	10.1		
	Radius	6.5	41.7

**Table 11:** Characteristic parameters predicted by the DRN with TFS and CNN-1D models in comparison to the actual values for test scenarios selected from the measurement data.

Scenario ID	Model	*D*	*P*	*R*	Error (*D, P, R*) [mm]
1	True value	165	900	15	–
	DRN with TFS [This work]	159	863	19	6, 37, − 4
	CNN-1D	137	791	17	28, 109, − 2
2	True value	170	550	20	–
	DRN with TFS [This work]	148	520	20	22, 30, 0
	CNN-1D	127	627	17	43, − 77, 3
3	True value	160	770	10	–
	DRN with TFS [This work]	164	785	19	− 4, − 15, − 9
	CNN-1D	138	746	18	22, 24, − 8

## Conclusion

This work introduced a methodology for surrogate-assisted buried object characterization in terms of estimating characteristic parameters, specifically, radius, depth and lateral position, using several types of data structures. These include the commonly used data types of received reflected signals in the form of raw B-scans (2D data), the extracted features from raw time signals such as PCs and SFs (mean, variance, standard deviation, skewness and kurtosis), as well as consecutive A-scans (1D data). The feature-based data sets are commonly used for classification approaches, and the feature-based dataset is employed to further investigate the performance of the proposed surrogate-assisted regression approach. The key performance figures of interest include the computational cost and reliability of the surrogate models in terms of the object characterization quality. It has been observed that the conventional network models using B-scan images require high computational cost and their performances in estimation parameters are not successful enough, so new variable data structures and deep learning surrogate models are investigated. In order to ensure the highest possible performance in the mentioned sense, a novel surrogate modeling approach with variable data structures and deep-learning-based framework has been introduced. Extensive investigations carried out for the aforementioned data structures revealed that the usage of B-scan (Case 1) is associated with high computational expenses, whereas the usage of extracted features (Case 2) reduces the model training time but deteriorates the object characterization quality. Consequently, the proposed approach focused on incorporating TFS data of consecutive raw A-scans (Case 3), handled and processed using DRN. The most important feature of the proposed modeling methodology includes computational efficiency as well as highly accurate predictions of the buried object parameters.

The proposed customized DRN model has been comprehensively trained and tested through TFS data obtained from a number of scenarios of cylindrical PEC object buried at different positions in various types of dispersive subsurface media. The proposed methodology has been compared with several benchmark models involving CNN-1D, SVRM, MLP, and GP regression. The outcomes with respect to the error metrics demonstrate superior performance of the proposed technique with the MAE as low as 3.1 mm against 11.4 mm for the best comparison technique, CNN-1D. Another outcome also demonstrates the requirement of low computational cost of the DRN with TFS framework. Thus, the proposed methodology can be considered as an accurate and computationally efficient approach to buried object characterization. For the sake of supplementary validation, the proposed DRN with TFS model has been tested using realistic scenarios with noisy data (SNR value of 20 dB and 30 dB), and the measurement data. Extensive numerical and experimental data, including error metrics, as well as comparisons between the model-predicted and actual parameters of the buried object for a number of test scenarios demonstrate practical utility of the introduced DRN with TFS model as well as its superiority over a family of state-of-the-art benchmarks models. On the other hand, the proposed methodology presents some limitations, in particular, characterization of objects featuring such as different material types and different shapes. The TFS data contains features from the time and frequency domain for subsurface medium and buried object. As mentioned, when the environment is more complex, the features of subsurface medium have the same effects on time frequency spectrogram for the entire scenarios of data set and the proposed surrogate model can learn to discriminate features of the buried object. Also, it can be used with a larger number of characteristic features in cascaded structures including classification and regression approaches together. Therefore, it should be emphasized that the proposed model is expandable for different subsurface media, material types and shapes. One of the objectives of the future work is to extend the applicability of the presented customized deep learning-based surrogate modeling technique for buried objects characterization in terms of featuring different material types and shapes.

## Data Availability

The datasets used and/or analysed during the current study available from the corresponding author on reasonable request.
